# Draft Genome Sequence of the Human-Pathogenic Bacterium *Vibrio alginolyticus* E0666

**DOI:** 10.1128/genomeA.00686-13

**Published:** 2013-08-29

**Authors:** Yuan Cao, Xiao-Fei Liu, He-Lin Zhang, Ying-Jian Chen, Cheng-Jin Hu

**Affiliations:** Department of Laboratory Medicine, Jinan Military General Hospital, Jinan, Shandong, China

## Abstract

*Vibrio alginolyticus* is a Gram-negative halophilic bacterium with worldwide distribution. In this work, we report the draft genome sequence of a *V. alginolyticus* strain (E0666) isolated from *Epinephelus coioides* ascites in the Shantou city of Guangdong Province, China.

## GENOME ANNOUNCEMENT

*Vibrio alginolyticus* is a Gram-negative halophilic bacterium with worldwide distribution (1). On the coast of southern China, *V. alginolyticus* is the dominant *Vibrio* species found both in seawater and in farmed marine animals (2, 3). Although *V. alginolyticus* is most commonly associated with wound infections, otitis media, and otitis externa (4, 5), it is increasingly recognized as an important intestinal pathogen in humans. In 1980, Hiratsuka et al. (6) isolated the first *V. alginolyticus* organism from a patient with acute enterocolitis. Recently, similar cases of *V. alginolyticus* infection that caused gastroenteritis or diarrhea have been reported (7–12). Consequently, there is a need to develop a rapid and accurate diagnostic method for the detection of *V. alginolyticus.*

*V. alginolyticus* strain E0666 was isolated from spoiled horse mackerel that had caused food poisoning and that was kindly provided by the Marine Culture Collection of China (Xiamen, Fujian Province, China). *V. alginolyticus* E0666 was grown overnight in alkaline peptone water (APW) supplemented with 2% (wt/vol) NaCl at 37°C with vigorous shaking. Bacterial cells were pelleted by centrifugation and the supernatants were discarded. The bacterial cells were washed three times with phosphate-buffered saline (PBS) before being resuspended to a final approximate concentration of 5 × 10^8^ CFU/ml. The QIAamp DNA mini kit (Qiagen) was used for DNA extraction. The quality and quantity of DNA were evaluated spectrophotometrically with the NanoDrop ND-1000 (Thermo Scientific, Wilmington, DE). A concentration of 50 ng/µl was used for next-generation sequencing.

*V. alginolyticus* E0666 was sequenced at the Chinese National Human Genome Center (CNHGC) in Shanghai using a massively parallel pyrosequencing technology (Roche 454 GS FLX), with a shotgun library of 1- to 1.4-kb insertion size. One hundred twenty-nine contigs (>500 bp) with a total size of 5,048,917 bp were assembled from 167,945 reads using the Newbler software of the 454 suite package, providing 19-fold coverage. The average contig length is 39,138 bp and the maximum contig length is 259,548 bp, with the overall G+C content of the *V. alginolyticus* genome assemblies being 44.8%. The sequencing accuracy of the draft genome was 99.9978%. The prediction and annotation of genes were done using the Rapid Annotations using Subsystems Technology (RAST) server (13). Functional annotation was also performed by PGAAP using the public database of National Center for Biotechnology Information (NCBI). All the contig sequences were searched against the NCBI Nucleotide database, and no allochthonous microorganism contamination was observed. The assembled genome of *Vibrio alginolyticus* comprises a single circular chromosome (4.9 Mbp, 44.8% G+C content), with 4,565 coding DNA sequences (CDSs), 8 rRNAs, and 82 tRNA sequences.

For the first time, we have obtained the whole-genome sequence of *V. alginolyticus* E0666. We hope this genome information provides positive contributions to the study of virulence factors and the development of new accurate diagnostic methods.

### Nucleotide sequence accession numbers.

This whole-genome shotgun project has been deposited at DDBJ/EMBL/GenBank under the accession no. AMPD00000000. The version described in this paper is the first version, AMPD01000000.
